# Nanoparticle-based biosensor integrated with CRISPR/Cas12b platform for sensitive and visual identification of hepatitis B virus pregenomic RNA in chronic hepatitis B patients

**DOI:** 10.1186/s12866-026-04900-4

**Published:** 2026-03-24

**Authors:** Xu Chen, Yanyan Qin, Shilei Dong, Cencen Jia, Ya Li, Yalan Liu, Qi Zhao, Qingxue Zhou

**Affiliations:** 1 The Second Clinical College, Guizhou University of Traditional Chinese Medicine, Guiyang, Guizhou 550003 People’s Republic of China; 2 Medical Science Laboratory of Integrative Chinese and Western Medicine, The Second Affiliated Hospital, Guizhou University of Traditional Chinese Medicine, Guiyang, Guizhou 550003 People’s Republic of China; 3 Department of Clinical Laboratory, Zhejiang Hospital, Hangzhou, Zhejiang 310013 People’s Republic of China; 4 Department of Gastroenterology, The Second Affiliated Hospital, Guizhou University of Traditional Chinese Medicine, Guiyang, Guizhou 550003 People’s Republic of China; 5 Clinical Laboratory, Hangzhou Women’s Hospital, Hangzhou, Zhejiang 310008 People’s Republic of China

**Keywords:** Chronic hepatitis B, Pregenomic RNA, CRISPR/Cas12b, Nanoparticle-based biosensor, Point-of-care testing, Loop-mediated isothermal amplification

## Abstract

**Background:**

Chronic hepatitis B (CHB) represents a leading driver of hepatocellular carcinoma and end-stage liver disease. Serum hepatitis B virus pregenomic RNA (HBV-pgRNA) has emerged as a new bioindicator strongly related to the efficacy and prognosis of CHB treatment. Seeking ultrasensitive, rapid, highly specific, and straightforward HBV-pgRNA detection, we constructed an innovative CRISPR-HBV-pgRNA platform through the integration of a CRISPR/Cas12b system with loop-mediated isothermal amplification (LAMP). Then, we interpreted the detection results via either real-time fluorescence (RTF) or a gold nanoparticle-based lateral flow biosensor (AuNPs-LFB).

**Methods:**

Herein, the AuNPs-based biosensor used was manufactured following our design. The unique LAMP primers and guide RNA (gRNA) were designed against the HBV-pgRNA gene, ensuring optimized diagnostic conditions: Reaction temperature and time. Both assay sensitivity and specificity were validated, and the feasibility was validated via clinical specimens from patients having chronic HBV infection.

**Results:**

The developed AuNPs-based biosensor was successfully fabricated. Primers LAMP and gRNA were specifically designed to target the HBV pgRNA sequence. The integrated assay protocol, comprising RNA extraction (45 min), RT-LAMP amplification (30 min), CRISPR/Cas12b cleavage (5 min), and visual readout (2 min), was completed in 85 min without reliance on costly instrumentation. The method achieved a detection limit of 10 copies/reaction and demonstrated no cross-reactivity with other tested pathogens.

**Conclusions:**

The CRISPR-HBV-pgRNA assay is a powerful diagnostic tool and exhibits considerable potential for POC testing for the evaluation of chronic HBV infection status and antiviral drug efficacy, especially for resource-limited regions.

**Supplementary Information:**

The online version contains supplementary material available at 10.1186/s12866-026-04900-4.

## Introduction

Chronic hepatitis B (CHB) represents a considerable global public health burden, with the potential to progress to cirrhosis and hepatocellular carcinoma (HCC) [1, 2]. Approximately 2 billion people were infected with the hepatitis B virus (HBV), of whom above 296 million live with chronic infection, leading to approximately 820,000 deaths annually from HBV-related complications [3, 4]. CHB frequently co-occurs with human immunodeficiency virus (HIV) and hepatitis C virus (HCV), amplifying the public health and socioeconomic burden, particularly in low-resource settings [5]. In response, the WHO set a global target in 2016 to eliminate viral hepatitis as a public health threat by 2030 [6]. For achieving this goal, simple and accurate diagnostic approaches are critical in HBV infection treatment and control. Intrahepatic HBV covalently closed circular DNA (cccDNA) represents a valuable biomarker for assessing treatment response in CHB, as it has the capacity to generate progeny viral DNA and proteins even when HBV DNA or HBsAg is undetectable in the peripheral blood [7]. However, using HBV cccDNA as a biomarker in real-world clinical practice presents a significant obstacle owing to the invasive nature of liver biopsy [8]. Serum HBV pregenomic RNA (pgRNA) is transcribed from cccDNA in infected hepatocytes, has been proposed as a possible intrahepatic cccDNA surrogate marker in CHB patients and during nucleos(t)ide analogs therapy [9–11].

An ideal pathogen diagnostic approach would be rapid, specific, sensitive, inexpensive, easy-to-operate, without expensive equipment, and delivered to the user according to the WHO ASSURED criteria (Affordable, Sensitive, Specific, User-friendly, Robust and rapid, Equipment-free, Deliverable to end users) [12]. Currently, PCR-based methods are widely used for detecting HBV-pgRNA clinically because of their high sensitivity and specificity [13, 14]. However, their utility is limited in resource-limited regions, where access to expensive thermal cyclers, specialized laboratory infrastructure, and trained personnel is often lacking. Isothermal amplification techniques: Loop-mediated isothermal amplification (LAMP) and recombinase polymerase amplification (RPA) offer a promising alternative by enabling nucleic acid (NA) amplification under isothermal conditions, thus eliminating the requirement for thermal cycling equipment. Despite their advantages, these methods suffer from intrinsic limitations in analytical specificity [15]. Therefore, there is a critical need for a highly sensitive, specific, cost-effective, and user-friendly point-of-care (POC) assay for HBV-pgRNA detection to support timely screening and management of CHB.

Recently, prokaryote-derived clustered regularly interspaced short palindromic repeat and CRISPR-associated protein (CRISPR/Cas) systems have become a transformative platform for next-generation, on-site NA detection, leveraging the collateral *trans*-cleavage activity of Cas effectors: Cas12a, Cas12b, Cas13a, and Cas14 [16–18]. When integrated with various isothermal amplification techniques, the CRISPR/Cas system has enabled the development of numerous highly sensitive, on-site NA detection platforms: CRISPR/Cas12b-based HOLMESv2, CRISPR/Cas13a-based SHERLOCK, and CRISPR/Cas12a-based DETECTR approaches [19–21]. Cas12b, a classical type of V-B CRISPR-Cas endonuclease, which can cleave target double-stranded DNA (dsDNA) by using a specific gRNA [19]. Meanwhile, its *trans*-cleavage activity was released and non-specifically degrades surrounding single-stranded DNA (ssDNA) [19]. The non-target ssDNA is labeled with either a fluorescent or an immune-based reporter. Cleavage of the labeled ssDNA then enables detection either via a fluorescent signal or through a gold nanoparticle-based lateral flow biosensor (AuNPs-LFB), respectively [22]. At POC detection, a paper-based biosensor using AuNPs on a lateral flow platform is widely employed owing to its high accuracy and reliability, ease of operation, and long-lasting stability [23, 24]. A detectable colorimetric shift occurs upon nanoparticle aggregation in the presence of the target analyte, allowing instrument-free result readout [24]. Inspired by the highly specific and ssDNA *trans*-cleavage activity of the Cas12b endonuclease, the CRISPR/Cas12b-based diagnostic platform has been successfully utilized to detect clinically relevant pathogens: *Mycobacterium tuberculosis*, HBV, and severe acute respiratory syndrome coronavirus-2 [25–27]. In the current study, CRISPR-Cas12b was integrated with LAMP and AuNPs-LFB to develop an innovative HBV-pgRNA POC detection system termed CRISPR-HBV-pgRNA. This method was developed to achieve accurate, sensitive, rapid, visual, and cost-saving HBV-pgRNA identification. The assay’s clinical feasibility was evaluated using specimens from patients with chronic HBV infection.

## Materials and methods

### Reagents and instruments

AapCas12b (C2c1) nuclease was obtained from Tolo Biotech (Shanghai, China). *Bst*-XT WarmStart DNA polymerase. DNase I, spin RNA Cleanup Kit, deoxynucleotide (dNTP) mix, and MgSO_4_ solution were purchased from New England Biolabs (Ipswich, USA). Avian myeloblastosis virus (AMV) reverse transcriptase was obtained from Invitrogen (Waltham, MA, USA). EasyPure Viral RNA Kit was sourced from TransGen Biotech Co., Ltd. (Beijing, China). AuNPs-LFB was sourced from HuiDeXin Biotechnology (Tianjin, China) per our design specifications. Abcam. Co., Ltd. (Shanghai, China) provided rabbit anti-fluorescein antibody (anti-FAM) and biotinylated bovine serum albumin (biotin-BSA). Streptavidin-coated AuNPs (SA-AuNPs; crimson red) (size, 34.46 ± 4.34 nm; extinction coefficient, 6.0 × 10^9^ M^− 1^ cm^− 1^ at 506 nm) were procured from Bangs Laboratories, Inc. (Indiana, USA). The AuNPs-LFB materials: Sample/absorbent/pad, nitrocellulose membrane (NC), and backing card, were provided by Jie-Yi Biotechnology. Co., Ltd. (Shanghai, China). Real-time turbidimeter (LA-500) was procured from Eiken Chemical Co., Ltd. (Tokyo, Japan).

### Clinical sample and standard panels preparation

Forty-six serum samples were gathered from CHB patients undergoing antiviral treatment. For the negative control group, twenty-four serum samples were obtained from healthy donors with no history of HBV infection. HBV RNA was extracted through the EasyPure Viral RNA Kit per protocol, and treated with DNase I at 37 °C for 10 min. Finally, the RNA was purified with the Spin RNA Cleanup Kit.

The National Standard for HBV RNA detection (Batch No. 340023 − 202001) was obtained from the National Institutes for Food and Drug Control.

### HBV-pgRNA LAMP primer and gRNA design

**Fig. 1:**
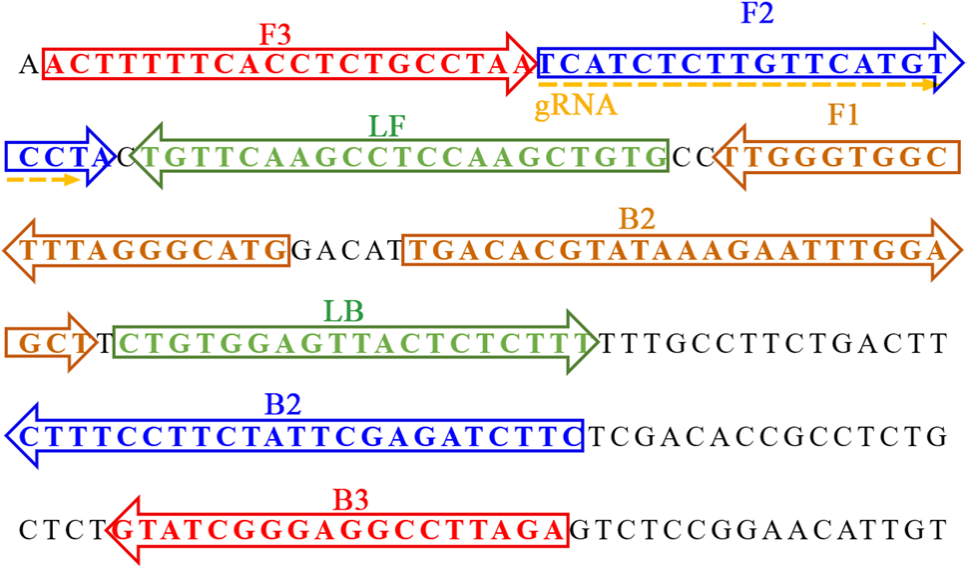
Sequences and location of HBV-pgRNA gene used to design CRISPR-HBV-pgRNA primers and gRNA. The sites of LAMP primers were in boxes, and the gRNA was underlined. Right arrows and left arrows indicated the sense and complementary sequences, which were used in this study, respectively

HBV-pgRNA LAMP primers were designed through online software Primer Explorer (v5.0; https://primerexplorer.eiken.co,jp/lampv5/index.html) and PRIMER PREMIER 5.0 per the LAMP mechanism targeting HBV pgRNA (GenBank accession no. LR745632.1). LAMP primer specificity was validated through the BLAST analysis. The gRNA for the HBV pgRNA was designed based on the CRISPR-Cas12b identification mechanism. Figure 1 depicts HBV-pgRNA LAMP primer and gRNA locations, and Table 1 provides LAMP primer and gRNA sequence details. Genscript Biotech Co., Ltd. (Nanjing, China) synthesized and purified all the oligonucleotides with HPLC purification grade.

**Table 1:** The primers and gRNAs used in the current study

Primers/gRNA	Sequence^a^	Length^b^	Target gene and nucleotide numbering
F3	5ʹ-ACTTTTTCACCTCTGCCTAA-3ʹ	20 nt	pgRNA Genbank Accession No. LR745632.1) (102–311)
B3	5ʹ-TCTAAGGCCTCCCGATAC-3ʹ	18 nt	
FIP*	5ʹ- CATGCCCTAAAGCCACCCAA-**TTC**TCATCTCTTGTTCATGTCCTA − 3ʹ	44 mer	
BIP	5ʹ- TGACACGTATAAAGAATTTGGAGCT-GAAGATCTCGAATAGAAGGAAAG − 3ʹ	48 mer	
LF	5ʹ- CACAGCTTGGAGGCTTGAACA-3ʹ	21 nt	
LB	5ʹ-CTGTGGAGTTACTCTCTTT-3ʹ	19 nt	
gRNA	5ʹ-GUCUAGAGGACAGAAUUUUUCAACGGGUGUGCCAAUGGCCACUUUCCAGGUGGCA AAGCCCGUUGAGCUUCUCAAAUCUGAGAAGUGGCACUCAUCUCUUGUUCAUGUCCU-3ʹ	111 mer	

^a^HBV-pgRNA-FIP* primers were modified in linker region with a PAM site (TTC)

^b^nt, nucleitide; mer, monomeric unit

### AuNPs-LFB preparation

**Fig. 2:**
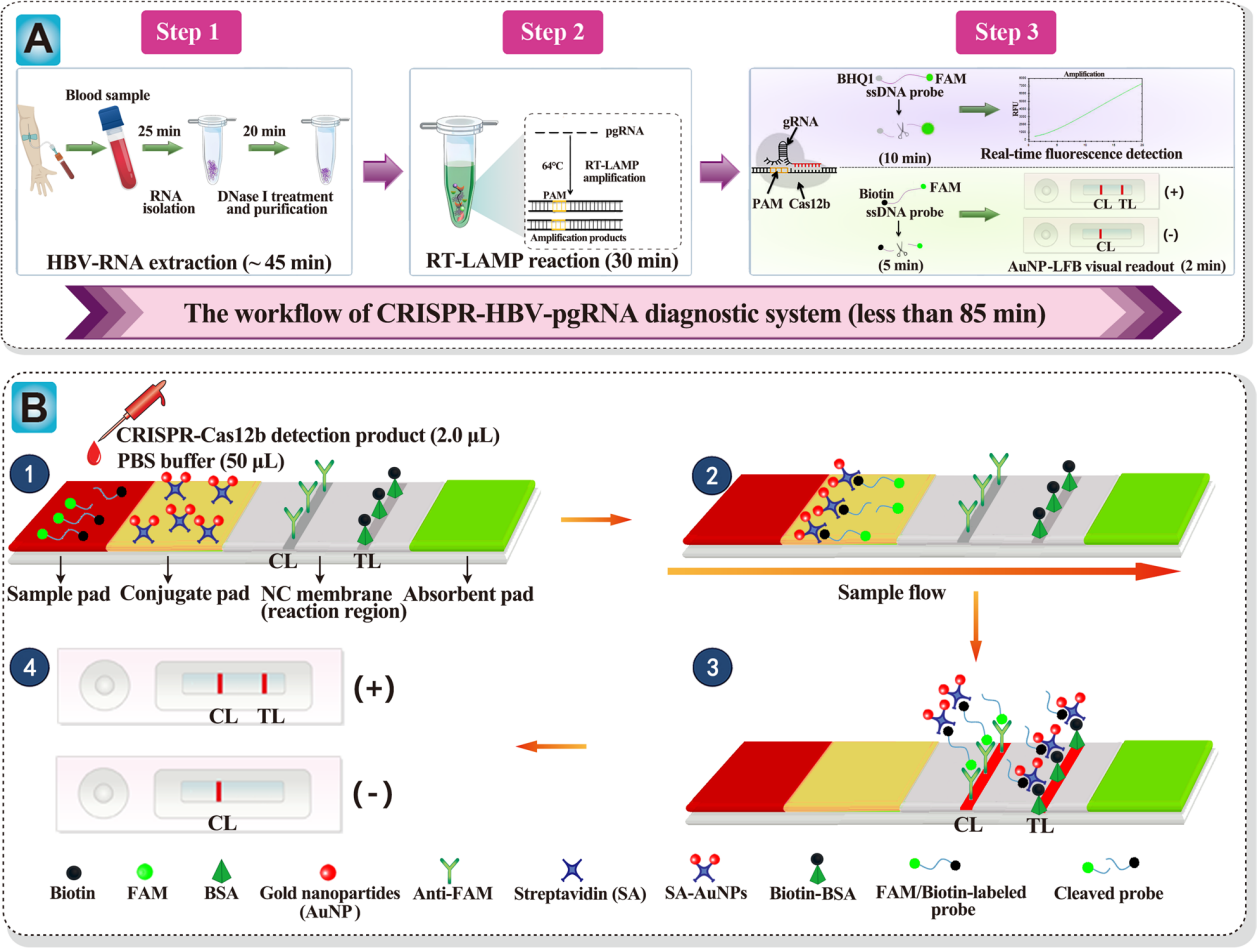
Schematic diagram of the CRISPR-HBV-pgRNA assay’s principle. **A** Workflow of the CRISPR-HBV-pgRNA assay. The entire workflow employs the following closely linked steps: the HBV RNA was extracted and treated with DNase I (step 1). The target gene HBV-pgRNA containing a PAM site (TTC) is specifically amplified by the RT-LAMP reaction (step 2). Upon recognition of the matching target sequence, the CRISPR/Cas12b complex was activated to non-specifically cleave a single-stranded DNA (ssDNA) probe. The CRISPR/Cas12b detection and their results were read out with real-time fluorescence (REF) or AuNPs-LFB methods (step 3). **B** Schematic diagram showing AuNPs-LFB principles for the visual interpretation of CRISPR-HBV-pgRNA outcomes. (1) CRISPR-HBV-pgRNA detection products (2.0 μL) and running buffer (50 μL) were simultaneously dropped on the sample pad. (2) The running buffer containing detection products moved forward onto the conjugate pad and NC membrane. Meanwhile, the dye SA-AuNPs was rehydrated with running buffer on the conjugate region. The biotin-labeled probes were integrated with SA-AuNPs at the conjugate pad. (3) For a positive result, the ssDNA probes (5'-FAM-TTTTTT-Biotin-3') were trans-cleaved by activated CRISPR/Cas12b nuclease, and the FAM and biotin were separated. And then, the biotin-SA-AuNPs complexes were captured by biotin-BSA at the TL. For a negative outcome, the ssDNA probes cannot be cleaved, and the FAM/Biotin-ssDNA-SA-AuNPs complexes were arrested by anti-FAM at the CL. **D** Interpretation of the CRISPR-HBV-pgRNA results. For positive outcomes, CL and TL appeared simultaneously on the AuNPs-LFB. For negative results, only the CL line was observed on the AuNPs-LFB

Figure 2B represents the AuNPs-LFB schematic. Briefly, the biosensor (size: 60 × 4 mm) comprises four functional components sequentially assembled on a plastic backing: Sample/conjugate pad, NC, and absorption pad. The SA-AuNPs was deposited onto the conjugate pad. The NC was patterned with two distinct lines: the control line (CL), fixed with Anti-FAM (4 mg/mL), and the test line (TL), immobilized with biotin-BSA (0.2 mg/mL), respectively. The two lines were spatially separated at a 5-mm interval to ensure an unambiguous signal readout. The AuNPs-LFB strip was manufactured through Tianjin HuiDeXin Biotech Co., Ltd. (Tianjin, China) per our design. The biosensors were maintained at room temperature before usage.

### CRISPR-HBV-pgRNA working principle

HBV-pgRNA-LAMP pre-amplification was employed in a 25 µL reaction volume. The reaction mixture comprised: 1 µL of *Bst*-XT WarmStart DNA polymerase (8 U), 1 µL of AMV reverse transcriptase (10 U), 2.5 µL of 10× reaction buffer, 1.5 µL of MgSO₄ (100 mM), 3.5 µL of dNTP mix (10 mM), 0.4 µM each of outer primers F3 and B3, 1.6 µM each of inner primers FIP* and BIP, 0.8 µM each of loop primers LF and LB, and 1 µL of NA template, with nuclease-free double-distilled water (ddH₂O) introduced to reach the final volume. Amplification was performed isothermally using a heat block. Seeking the determination of the optimal reaction temperature, we monitored real-time turbidity using a LA-500 turbidimeter.

Here, the AapCas12b (C2c1) was deployed for CRISPR-Cas-based *trans*-cleavage detection. First, the CRISPR-Cas12b-gRNA complexes were pre-assembled in a 50 µL reaction volume, containing 2 µL AapCas12b (10 µM), 2 µL gRNA (10 µM), and 5.0 µL 10×HOLMES buffer, adding nuclease-free water to the volume. The mixture was preincubated at 37°C for 10 min and utilized instantly or maintained at 4°C for no more than 24 h. The *trans*-cleavage reaction was carried out in a 25 µL system consisting of 4 µL pre-assembled Cas12b–gRNA complex, 2 µL HBV-pgRNA LAMP amplicon, 2.5 µL 10× HOLMES buffer, 1 µL ssDNA reporter, and nuclease-free water to a final volume. The reaction was subjected to incubation at 45°C for 5 min. Detection was performed simultaneously via real-time fluorescence (RTF) and AuNPs-LFB methods. For RTF detection, we utilized the Flu-probe (5’-FAM-TTTTTT-BHQ1-3’, 10 µM) as ssDNA reporter, while placing the ssDNA probe with 5’-FAM-TTTTTT-Biotin-3’ (10 µM) for the AuNPs-LFB assay.

### CRISPR-HBV-pgRNA assay sensitivity and specificity

To evaluate the assay sensitivity, HBV RNA standards were serially diluted ranging from 1.0 × 10^5^ to 1 copy/reaction. The CRISPR-HBV-pgRNA reactions were carried out as outlined earlier, analyzing the results with RTF and AuNPs-LFBm and testing each dilution at least three times at differently days.

Our assay specificity was tested through HBV RNA standards, clinical HBV RNA isolates from CHB patient serum, and other microbial NA templates (Table 2). Herein, the microbial NA templates were isolated through Bacterial Genomic DNA Extraction Kits or Virus DNA/RNA Extraction Kits (Xi’an Tianlong Technology Co., Ltd.; Xi’an, China), measuring each sample concentration (> 5.0 × 10^4^ copies/mL) through a NanoDrop ND-2000 spectrophotometer (Beijing, China) at A260/280. The detection process was carried out as outlined earlier, simultaneously analyzing the results with RTF and AuNPs-LFB, performing all tests in at least three replicates at differently days.

**Table 2:** Microbial strains used in the current study

NO.	Strains/Templates	Source of strains^a^	No. of strains	CRISPR-HBV-pgRNA assay^b^	
				AuNP-LFB	RTF
1	HBV-RNA (standard substance)	National Institutes for Food and Drug Control	1	P	P
2	HBV-RNA (clinical samples)	2nd GZUTCM	10	P	P
3	Hepatitis C virus	2nd GZUTCM	1	N	N
4	Human immunodeficiency virus	GZCCL	1	N	N
5	Human papilloma virus	GZCCL	1	N	N
6	Human rhinovirus	2nd GZUTCM	1	N	N
7	Influenza A virus	2nd GZUTCM	1	N	N
8	Influenza B virus	2nd GZUTCM	1	N	N
9	Epstein-Barr virus	2nd GZUTCM	1	N	N
10	Coxsackie virus CAV16	2nd GZUTCM	1	N	N
11	Human enterovirus EV71	2nd GZUTCM	1	N	N
12	Herpes zoster virus	Hangzhou Women’s Hospital	1	N	N
13	Adenoviridae	Hangzhou Women’s Hospital	1	N	N
14	*Escherichia coli*	ATCC8739	1	N	N
15	*Staphylococcus aureus*	ATCC25923	1	N	N
16	*Haemophilus influenzae*	ATCC49247	1	N	N
17	*Klebsiella pneumoniae*	ATCC700603	1	N	N
18	*Pseudomonas aeruginosa*	ATCC27853	1	N	N
19	*Neisseria gonorrhoeae*	Hangzhou Women’s Hospital	1	N	N
20	*Chlamydia trachomatis*	Hangzhou Women’s Hospital	1	N	N

^a^2nd GZUTCM, the Second Affiliated Hospital, Guizhou University of Traditional Chinese Medicine, GZCCL, Guizhou Provincial Center for Clinical Laboratory, *ATCC* American Type Culture Collection

^b^*P* Positive, *N* Negative

## Verification of CRISPR-HBV-pgRNA assay feasibility for clinical samples

Here, 46 serum samples from CHB patients and 24 serum specimens from healthy donors were utilized to verify our assay feasibility. The virus RNA was extracted through the EasyPure Viral RNA Kit and treated with DNase I at 37 °C for 10 min. The RNA was purified with the Spin RNA Cleanup Kit, thereby performing the CRISPR-HBV-pgRNA assay as outlined earlier. All serum samples were tested through commercially available RT TaqMan PCR Kits for HBV RNA and HBV DNA (SanSure Biotech; Changsha, China) through an Applied Biosystems™ 7500 Real-Time PCR System (Life Technologies; Singapore). Measurements of HBV DNA > 5 IU (~ 30 copies/mL) and HBV RNA > 50 copies/mL represented a positive result per the protocols. Finally, CRISPR-HBV-pgRNA assay results were compared and analyzed against those of HBV-DNA- or HBV-RNA-real-time PCR approaches through online statistical software MedCalc [28].

## Results

### CRISPR-HBV-pgRNA diagnostic system overview

Figure 2A presents the CRISPR-HBV-pgRNA diagnostic system workflow and underlying principles. Briefly, HBV RNA was isolated and treated with DNase I as previously described **(**Fig. 2A, step 1). Then, the HBV-pgRNA was amplified via RT-LAMP for half an hour at 64 °C (Fig. 2A, step 2). The HBV-pgRNA-LAMP amplicons were designed to include the Cas12b PAM site, enabling specific recognition by the CRISPR-Cas12b system (Fig. 2A; step 3). Through gRNA, Cas12b binds to the target sequence, which activates its trans-cleavage activity, leading to rapid degradation of ssDNA reporter probes (Fig. 2A; step 3). Signal detection was performed in parallel via RTF with the Flu-probe and AuNPs-LFB with ssDNA probes. The detection process was completed in 85 min.

**Fig. 3:**
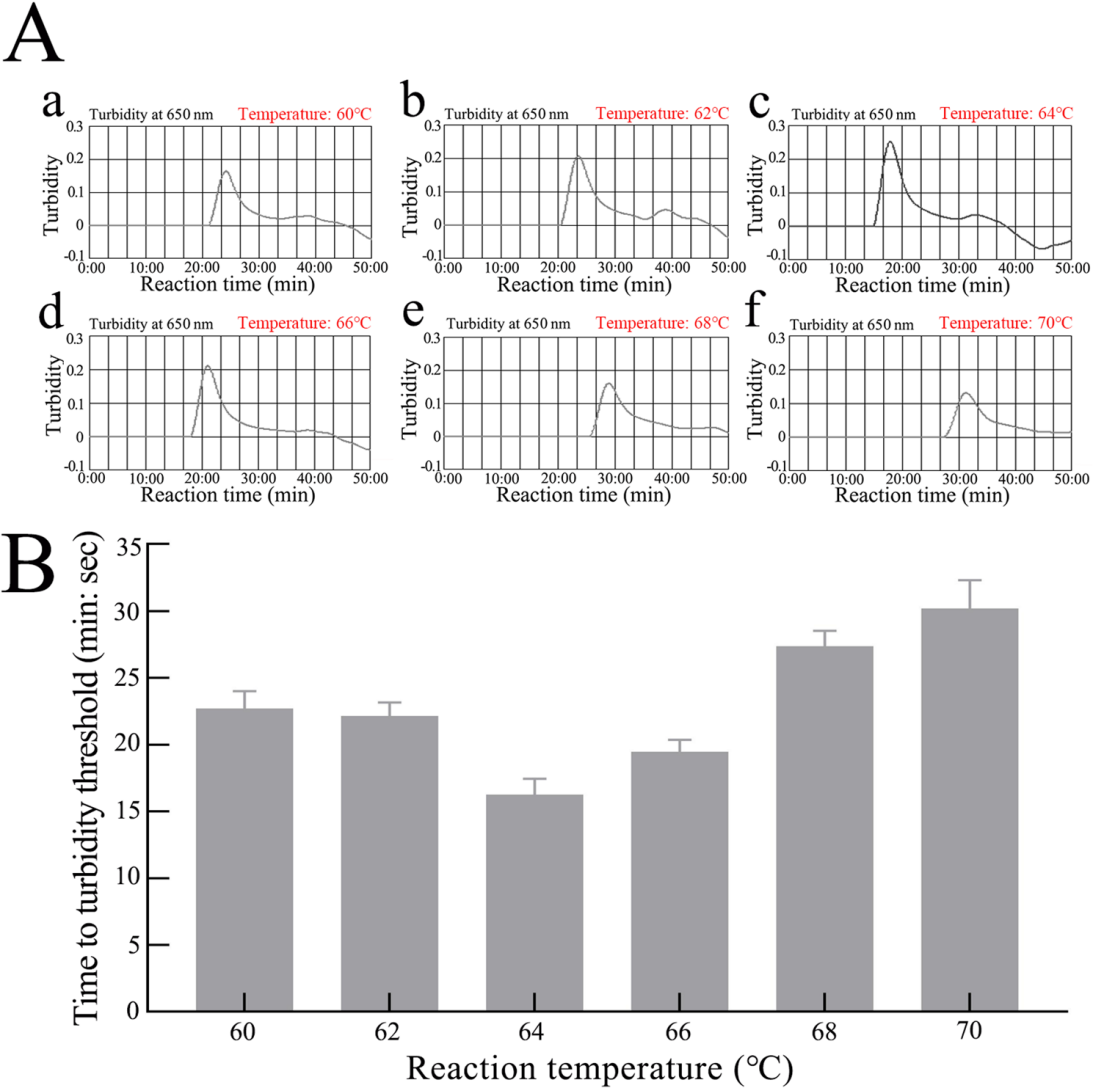
Optimizing the temperature for the LAMP pre-amplification stage in the CRISPR-HBV-pgRNA detection system. **A** The LAMP amplifications for the detection of HBV-pgRNA were monitored using real-time turbidity, and the corresponding curves of amplicons were displayed in the graphs. Turbidity > 0.1 indicated a positive value. Six kinetic graphs (**a**-**f**) were obtained at different temperatures (60–70 °C, 2 °C intervals) with 1 × 104 copies of the target gene . **B** Optimization of reaction temperature for HBV-pgRNA-RT-LAMP. The robust and efficient HBV-pgRNA-RT-LAMP amplification occurred at 64 °C

**Fig. 4:**
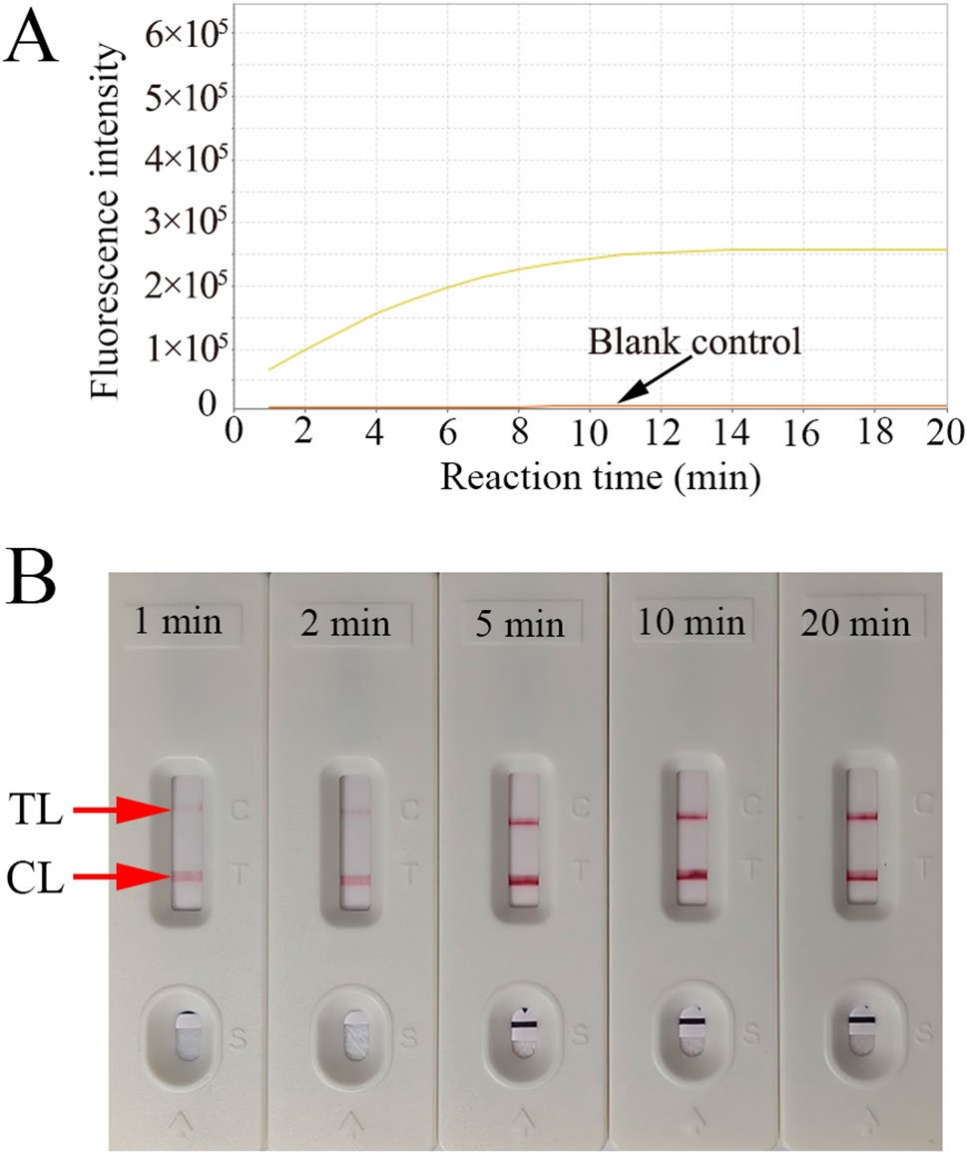
Optimizing the reaction time for CRISPR-Cas12b/gRNA cleavage. LAMP products (2 µL), yielded from 10 copies of HBV-RNA standard sub-stance, were added to the corresponding CRISPR-Cas12b/gRNA detection. The robust ﬂuorescent signal was detected within 10 min (**A**), and the re-markable signal at the test line (TL) of AuNPs-LFB appeared within 5 min (**B**)

For AuNPs-LFB interpretation, HBV-pgRNA-LAMP-CRISPR-Cas12b detection products (2.0 µL) and running buffer (50 µL; 100 mM PBS, pH 7.4) were applied simultaneously to an AuNPs-LFB sample pad (Fig. 2B**-**①). The mixture migrated along the strip via capillary action, rehydrating the lyophilized SA-AuNPs on the conjugate pad (Fig. 2B-②). In a positive reaction, the ssDNA probes were trans-cleaved via activated CRISPR-Cas 12b nuclease, separating the FAM and biotin moieties. Therefore, the biotin-SA-AuNPs complexes were captured by biotin-BSA at TL (Fig. 2B-③). In a negative reaction, the ssDNA probes remained linked to both FAM and biotin; thus, the SA-AuNPs-ssDNA probes were specifically arrested by anti-FAM at CL (Fig. 2B-③). Figure 2B-④ outlines the interpretation of the CRISPR-HBV-pgRNA detection through AuNPs-LFB.

### CRISPR-HBV-pgRNA assay optimal reaction conditions

The temperature optimization of the LAMP pre-amplification stage in the CRISPR-HBV-pgRNA detection system was investigated from 60 to 70℃ with HBV RNA standards (1.0 × 10^4^ copies). The HBV-pgRNA-LAMP reactions were monitored with real-time turbidimetry (LA-500), thereby plotting the kinetics graphs corresponding to the diverse temperatures (Fig. 3Aa-f), and the reaction time to threshold values were showed in Fig. 3B. The results suggested that robust and efficient HBV-pgRNA-LAMP amplification occurred at 64 °C (Fig. 3A-B).The CRISPR-Cas12b detection stage optimal reaction time was tested through multiple reaction times (1, 2, 5, 10, and 20 min). AuNPs-LFB and RTF were simultaneously applied to analyze the outcomes, indicating that a robust fluorescent signal was presented within 10 min, and a stable visual signal of AuNPs-LFB appeared within 5 min (Fig. 4A and B).

### CRISPR-HBV-pgRNA assay sensitivity and specificity

**Fig. 5:**
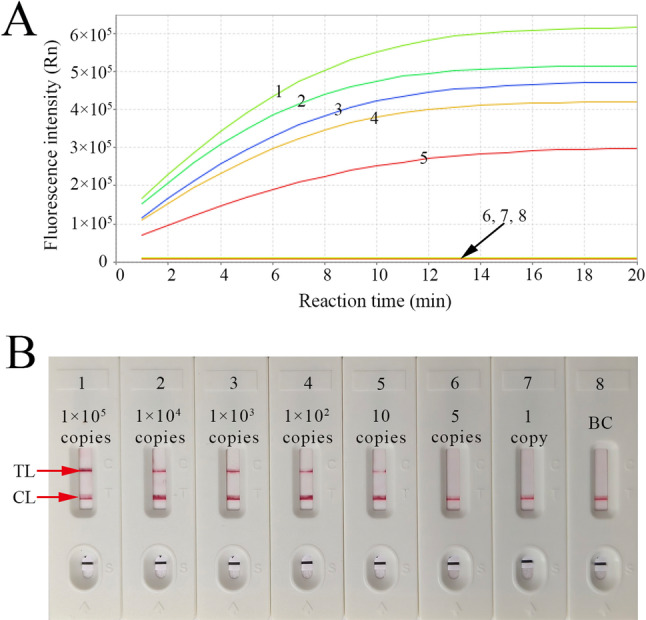
Sensitivity of the CRISPR-HBV-pgRNA assay. **A** real-time fluorescence (RTF) and (**B**) AuNPs-LFB approaches were simultaneously used to analyze the CRISPR-HBV-pgRNA outcomes. **A**/**B** 1–8 represented the HBV-RNA standard substance concentrations 1 × 10^5^, 1 × 10^4^, 1 × 10^3^, 1 × 10^2^, 10, 5, 1 copies/test and ddH_2_O, respectively. The limit of detection (LoD) of the CRISPR-HBV-pgRNA diagnostic system was 10 copies per reaction. CL: control line; TL: test line. “+”: positive; “—”: negative

To evaluate systematically the CRISPR-HBV-pgRNA assay limitation of detection (LoD), serial dilutions of HBV RNA standards ranging from 1.0 × 10^5^ to 1 copy were tested at the optimal detection conditions (HBV-pgRNA-LAMP amplification at 64 °C for 30 min, and CRISPR-Cas12b at 45 °C for 5 min), analyzing the results with RTF and AuNPs-LFB methods. The LoD of our assay was 10 copies/test (Fig. 5A and B). Strikingly, the visual RO of AuNPs-LFB aligned with RTF detection (Fig. 5A and B).

**Fig. 6:**
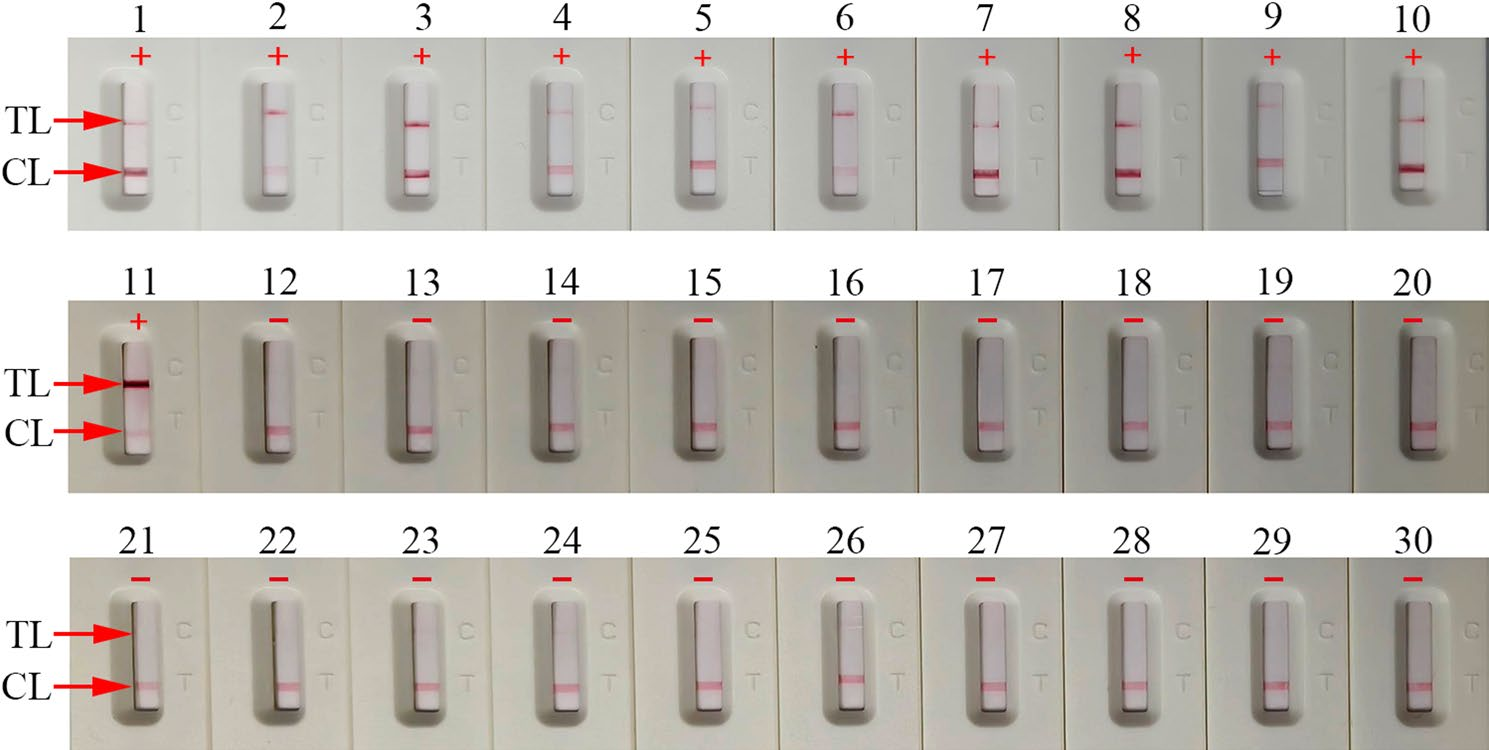
The specificity analysis of the CRISPR-HBV-pgRNA assay with different strains. The CRISPR-HBV-pgRNA assay was performed with different NA templates. Biosensor 1, HBV-RNA standard substance; biosensor 2–11, ten HBV-RNA positive clinical serums (confirmed with HBV-RNA real-time qPCR); biosensor 12–30, HCV, HIV, Human papilloma virus, Human rhinovirus, Influenza A virus, Influenza B virus, Epstein-Barr virus, Coxsackie virus CAV16, Human enterovirus EV71, Herpes zoster virus, Adenoviridae, *Escherichia coli*, *Staphylococcus aureus*,* Haemophilus influenzae*,* Klebsiella pneumoniae*,* Pseudomonas aeruginosa*,* Neisseria gonorrhoeae*,* Chlamydia trachomatis*, ddH_2_O (blank control), respectively. CL: control line; TL: test line. “+”: positive; “—”: negative

CRISPR-HBV-pgRNA assay specificity was tested using HBV-RNA standard, HBV-RNA positive clinical serums (validated by HBV RNA real-time TaqMan PCR), and 18 other microbial NA templates (Table 2). As expected, only the HBV-RNA templates exhibited positive outcomes (Fig. 6 and Fig. S1), indicating that the CRISPR-HBV-pgRNA assay can identify HBV-pgRNA with high specificity, with no cross-reactions.

### Evaluating CRISPR-HBV-pgRNA assay feasibility using clinical samples

To evaluate the CRISPR-HBV-pgRNA assay as a valuable HBV-RNA identification tool, 46 serum samples from CHB patients (undergoing antiviral treatment) and 24 serum specimens from healthy donors were tested simultaneously through three approaches, including HBV-RNA-qPCR, HBV-DNA-qPCR, and CRISPR-HBV-pgRNA. All 46 CHB samples were correctly identified as positive via HBV-RNA-qPCR, which aligned with those of our CRISPR-HBV-pgRNA assay. However, the HBV-DNA-qPCR results only identified 44 CHB samples as positive. Moreover, all three methods diagnosed correctly the 24 serum specimens from healthy donors as negative **(**Table 3, S1, Figs. S2–3). Unlike the HBV-RNA-qPCR technique, our assay’s sensitivity and specificity were 100% (95% CI: 92.29 to 100.00%) and 100% (95% CI: 85.75 to 100.00%). However, the sensitivity and specificity of HBV-DNA-qPCR were 95.65% (95% CI: 85.16 to 99.47%) and 100% (95% CI: 85.75 to 100.00%; Table 3). In addition, readout visuals AuNPs-LFB aligned with RTF in the CRISPR-HBV-pgRNA diagnostic system. Altogether, the CRISPR-HBV-pgRNA assay represented an advanced diagnostic tool for identifying CHB infection, especially in the antiviral treatment stage.

**Table 3:** Comparison of CRISPR-HBV-pgRNA diagnostic system, HBV-RNA-qPCR, and HBV-DNA-qPCR methods to detect chronic HBV infection in clinical samples

Detection assay	Results	Reference method(HBV-RNA-qPCR)		Sensitivity		Specificity	
		+	-	Value	95% CI	Value	95% CI
HBV-DNA-qPCR	+	44	0	95.65%	85.16 to 99.47%	100.00%	85.75 to 100.00%
	-	2	24				
CRISPR-HBV-pgRNA assay	+	46	0	100.00%	92.29 to 100.00%	100.00%	85.75 to 100.00%
	-	0	24				

## Discussion

Herein, A novel CRISPR-HBV-pgRNA diagnostic platform was successfully developed by integrating CRISPR-Cas12b-mediated detection with LAMP-based pre-amplification, enabling ultrasensitive, specific, and visual detection of serum HBV pgRNA. The clinical assay feasibility was validated via serum samples from patients having chronic HBV infection, and results were compared with those obtained by qPCR for both HBV-DNA and HBV-RNA levels.

HBV, a partly dsDNA virus, belongs to the *Hepadnaviridae* family with various serological and molecular markers, such as hepatitis B surface antigen (HBsAg) and anti-HBs, anti-HBc IgM and IgG, HbeAg, anti-HBe, HBV-DNA cccDNA, and HBV-RNA [29, 30]. Acute or chronic HBV infection diagnosis is established by testing these serological markers in serum, typically using ELISA or chemiluminescence immunoassay [31]. Nevertheless, these immunoassays are susceptible to false-negative results owing to the hook effect [32, 33]. In addition, the CHB patients may sustain low expression levels of HBsAg in peripheral blood because the HBV DNA can be integrated into the genome of HBV-infected hepatocytes [34]. Compared to serological approaches, NA amplification tests (NAATs) offer higher sensitivity and specificity with HBV DNA, cccDNA, and HBV RNA acting as targets for identification [35, 36]. Serum HBV DNA could not serve as a reliable biomarker because a level that falls below the limit of detection (LoD) does not necessarily imply the elimination of the virus, only that its reverse transcription is suppressed [34]. The cccDNA in the nucleus of infected hepatocytes is crucial in HBV infection chronicity and durability [7, 8, 34]. However, the detection of intrahepatic HBV cccDNA relies on liver biopsy, a method whose widespread clinical adoption is limited by its invasiveness, risk of complications, variable specimen yield, and inter-observer bias. Serum HBV-pgRNA, a transcript derived from viral cccDNA, has emerged as a promising biomarker that faithfully reflects intrahepatic cccDNA presence and transcriptional activity in patients, even if those receiving nucleos(t)ide analogs therapy [37, 38]. Many diagnostic methods for serum HBV-pgRNA primarily rely on PCR-based techniques. Gao et al. applied a one-step qRT-PCR assay and detected 100 copies/reaction for HBV-pgRNA agent [39]. RT-PCR and droplet digital PCR (ddPCR) were employed by Paturel et al. to evaluate a stable clonal cell line engineered to produce an RNA-based standard for calibrating circulating HBV RNA assays [14]. Nguyen et al. fabricated a novel selective RT-PCR assay for HBV pgRNA detection, which was verified with a LoD of 100 copies/mL [40]. However, the dependency of these techniques on sophisticated instrumentation and specialized personnel often renders them cost-prohibitive and inaccessible in resource-limited settings. In our previous study, we developed a RT-LAMP assay targeting the pgRNA of HBV, achieving a limit of detection LoD of 50 copies/mL [41]. Here, our integrated CRISPR-HBV-pgRNA platform enables isothermal amplification without specialized instruments, with results subsequently read out either visually by an AuNPs-based biosensor or via RTF. The entire detection process, from HBV RNA extraction to results interpretation, is efficiently completed within 85 min, which includes the sequential steps of RNA extraction (45 min), RT-LAMP (30 min), CRISPR/Cas12b detection (5 min), and results visual readout (2 min). We summarized the reported methods for HBV-pgRNA detection, their analytical properties were shown in Table 4.

**Table 4:** Comparison of the reported methods for HBV-pgRNA detection

Technology	Target	Limit of detection (LOD)	Total assay time	Operational complexity	Sample type	Reference
RT-qPCR	HBV pgRNA	100 copies/reaction	~ 2–3 h	High (thermal cycler required)	Serum	39
RT-qPCR	HBV pgRNA	100 copies/mL	~ 2–3 h	High (thermal cycler required)	Serum	40
ddPCR	HBV pgRNA	10 copies/mL	> 4 h	Very high (specialized instrumentation)	Serum	14
RT-LAMP assay	HBV-pgRNA	50 copies/mL	< 80 min	Low (isothermal, visual readout)	Serum	41
CRISPR/Cas12b-LAMP	HBV-pgRNA	10 copies/test	< 85 min	Low (isothermal, visual readout)	Serum	Current study

*RT-qPCR* real-time quantitative PCR, *ddPCR* droplet digital PCR, *RT-LAMP* reverse transcription loop-mediated isothermal amplification, *AuNPs-LFB* gold nanoparticle-based lateral flow biosensor

The CRISPR/Cas system, an adaptive immune mechanism in archaea and bacteria, is derived from the interplay of CRISPR sequences and Cas proteins [16–18]. It has been repurposed as a versatile, RNA-programmable platform for diverse applications, including genome editing, epigenetic regulation, transcriptional modulation, and NA detection [42]. Within the system, the CRISPR array, composed of spacers and repeats, serves to anchor exogenous sequences, whereas the Cas proteins facilitate the recognition and cleavage of exogenous gene fragments [18, 42]. The Cas effector proteins are guided by a pre-CRISPR RNA (crRNA) or a gRNA to execute precise cleavage at the specified location [43]. The gRNA-guided targeting mechanism ensures highly specific, target-dependent cleavage activity, significantly enhancing the system specificity [42, 43]. Recently, CRISPR/Cas systems have represented powerful tools for NA detection because of their precise sequence recognition and collateral *trans*-cleavage activity [44]. The CRISPR/Cas13a-based assay was used for HBV-DNA detection [45]. In this study, a specific gRNA was designed to target HBV pgRNA, effectively guiding the Cas12b effector to the intended sequence. The CRISPR-HBV-pgRNA assay specificity was evaluated via HBV RNA standards and a panel of unrelated pathogens, demonstrating no cross-reactivity (Fig. 6 and S1). Furthermore, the CRISPR-HBV-pgRNA diagnostic platform detected HBV pgRNA at concentrations as low as 10 copies/test (Fig. 5). Collectively, our novel CRISPR-HBV-pgRNA assay can accurately and sensitively identify HBV-pgRNA. Furthermore, we deployed the CRISPR-HBV-pgRNA assay to assess clinical serum samples from CHB patients and healthy donors. Unlike the HBV-RNA-qPCR technology, our assay’s sensitivity and specificity were 100% (95% CI: 92.29 to 100.00%) and 100% (95% CI: 85.75 to 100.00%). However, HBV-DNA-qPCR sensitivity and specificity were 95.65% (95% CI: 85.16 to 99.47%) and 100% (95% CI: 85.75 to 100.00%; Table 3). Taken together, the CRISPR-HBV-pgRNA assay represented an advanced diagnostic tool for identifying CHB infection, especially in the antiviral treatment stage.

Here, LAMP was used to pre-amplify the HBV-pgRNA target sequences. LAMP constitutes a robust isothermal NA amplification method that utilizes *Bacillus stearothermophilus* DNA polymerase with 4–6 primers targeting 6–8 unique regions of the gene, enabling efficient target sequence amplification at 58–69 °C constant temperature within 30 min [46, 47]. A standard LAMP primer set includes forward and reverse outer and inner primers, which are typically sufficient for amplification; however, the addition of loop primers enhances both reaction speed and specificity. Herein, we successfully fabricated an LAMP primer set to target the HBV-pgRNA gene, with optimal amplification conditions for the HBV-pgRNA-LAMP reaction being 64 °C for 30 min (Fig. 3A and B).

In our diagnostic platform, an AuNPs-LFB was utilized to achieve a visual readout of CRISPR-HBV-pgRNA outcomes. The AuNPs-LFB, being a paper-based biosensor, is well-suited for POC testing, attributing to its high sensitivity and specificity, ease of fabrication, low cost, user-friendly operation, and a direct visual RO [48, 49]. In our diagnostic design, the AuNPs-LFB can interpret visually the CRISPR-HBV-pgRNA outcome by employing an NC membrane pre-coated with anti-FAM at the CL and BSA-biotin at the TL for specific signal capture. Upon a positive result, the ssDNA reporter was *trans*-cleaved with the activated CRISPR-Cas12b, separating FAM and biotin probes, and capturing biotin-SA-AuNPs complexes by biotin-BSA at TL. For a negative result, the ssDNA reporters remain intact, and the FAM/biotin-labeled probe-SA-AuNPs complexes were arrested by the immobilized anti-FAM antibodies at CL. Herein, an RTF technique with Flu-probe was applied to analyze the CRISPR-HBV-pgRNA results. The total cost of each assay was estimated to be less than US $3.5, including HBV-RNA isolation (approximately US $1.0), LAMP (approximately US $0.3), CRISPR/Cas12b (0.5), AuNP-LFB detection (~ US $1.0).

Our study also has several limitations. First, the CRISPR-HBV-pgRNA assay developed in this study was primarily designed for the qualitative identification of HBV RNA. Interestingly, the real-time fluorescence data indicate its potential for semi-quantitative application, as a positive correlation was observed between fluorescence intensity and HBV RNA concentration (Fig. 5A). Therefore, establishing a robust, quantitative CRISPR-based method for HBV pgRNA detection constitutes a valuable and important direction for our future research. Second, the detection of HBV-RNA is challenging because the pgRNA is approximately 1.1 times the HBV genome length and overlaps entirely with the relaxed circular DNA sequence. To address this, pre-digestion of the extracted nucleic acid mixture with DNase I is an effective strategy to eliminate HBV-DNA interference [9, 14], nevertheless, the efficiency of DNase I digestion was not evaluated in this study, Future research should address this by incorporating appropriate controls to quantify digestion efficiency, thereby strengthening the experimental methodology and the reliability of the conclusions. More over, this multi-step procedure is time-consuming and should be further optimized in future work to streamline RNA template isolation. Third, the current assay necessitates opening the reaction tube between LAMP and CRISPR/Cas12b-based detection, thereby increasing the risk of carryover contamination. Developing a one-tube or closed-tube system would mitigate this aerosol risk and facilitate the method’s clinical translation.

## Conclusions

We have successfully fabricated an innovative diagnostic platform integrating LAMP pre-amplification with CRISPR-Cas12b, enabling the specific, sensitive, rapid, and visual HBV pgRNA identification. Our assay demonstrated a LoD as low as 10 copies/test and exhibited no cross-reactivity with other tested pathogens. The detection procedure, including HBV-RNA extraction (45 min), RT-LAMP (30 min), CRISPR/Cas12b detection, and readout of results (less than 10 min), was performed within 85 min, without requiring any specialized instrumentation. Therefore, our assay holds promise as a practical POC diagnostic tool to facilitate more accurate and convenient monitoring of chronic HBV infection status and assessment of antiviral therapeutic efficacy.

## Supplementary Information

Supplementary Material 1.

Supplementary Material 2.

Supplementary Material 3.

Supplementary Material 4.

Supplementary Material 5.

## Data Availability

The original contributions presented in the study are included in the article/Supplementary Material. Further inquiries can be directed to the corresponding authors.
