# Correction: Nanoparticle-based biosensor integrated with CRISPR/Cas12b platform for sensitive and visual identification of hepatitis B virus pregenomic RNA in chronic hepatitis B patients

**DOI:** 10.1186/s12866-026-05313-z

**Published:** 2026-07-09

**Authors:** Xu Chen, Yanyan Qin, Shilei Dong, Cencen Jia, Ya Li, Yalan Liu, Qi Zhao, Qingxue Zhou

**Affiliations:** 1 The Second Clinical College, Guizhou University of Traditional Chinese Medicine, Guiyang, Guizhou 550003 People’s Republic of China; 2 Medical Science Laboratory of Integrative Chinese and Western Medicine, The Second Affiliated Hospital, Guizhou University of Traditional Chinese Medicine, Guiyang, Guizhou 550003 People’s Republic of China; 3 Department of Clinical Laboratory, Zhejiang Hospital, Hangzhou, Zhejiang 310013 People’s Republic of China; 4 Department of Gastroenterology, The Second Affiliated Hospital, Guizhou University of Traditional Chinese Medicine, Guiyang, Guizhou 550003 People’s Republic of China; 5 Clinical Laboratory, Hangzhou Women’s Hospital, Hangzhou, Zhejiang 310008 People’s Republic of China

**Correction: BMC Microbiol 26, 485 (2026)**

**https://doi.org/10.1186/s12866-026-04900-4**

Following publication of the original article [1], the author reported that the captions for Figures 4 and 5 were swapped.

The original article has been updated.
