# Supplementary material for: Nanoparticle-based biosensor integrated with CRISPR/Cas12b platform for sensitive and visual identification of hepatitis B virus pregenomic RNA in chronic hepatitis B patients
Source: BMC Microbiol. 2026 Mar 24;26:485. doi: 10.1186/s12866-026-04900-4 (PMC13196004; doi:10.1186/s12866-026-04900-4)
Supplement: Supplementary file 1 — Supplementary Material 1. [file 12866_2026_4900_MOESM1_ESM.docx]

**Supplemental material**

**Nanoparticle-based biosensor integrated with CRISPR/Cas12b platform for sensitive and visual identification of hepatitis B virus pregenomic RNA in chronic hepatitis B patients**

Xu Chen^1,2Δ*^, Yanyan Qin^1,2Δ^, Shilei Dong^3^, Cencen Jia^1,2^, Ya Li^1,2^, Yalan Liu^1,2^, Qi Zhao^1,4*^, Qingxue Zhou^5*^

^1^The Second Clinical College, Guizhou University of Traditional Chinese Medicine, Guiyang, Guizhou, 550003, People′s Republic of China

^2^Medical Science Laboratory of Integrative Chinese and Western Medicine, the Second Affiliated Hospital, Guizhou University of Traditional Chinese Medicine, Guiyang, Guizhou, 550003, People′s Republic of China

^3^Department of Clinical Laboratory, Zhejiang Hospital, Hangzhou, Zhejiang 310013, People′s Republic of China

^4^Department of gastroenterology, the Second Affiliated Hospital, Guizhou University of Traditional Chinese Medicine, Guiyang, Guizhou, 550003, People′s Republic of China

^5^Clinical Laboratory, Hangzhou Women′s Hospital, Hangzhou, Zhejiang 310008, People′s Republic of China

^Δ^Drs. Xu Chen and Yanyan Qin have contributed equally to this work.

^*^Corresponding author:

Qingxue Zhou: [xuemmc@163.com](mailto:xuemmc@163.com)

Qi Zhao: 892551348@qq.com

Xu Chen: xuchen1220@126.com

**Table S1** Comparison of CRISPR-HBV-pgRNA diagnostic system, HBV-RNA-qPCR, and HBV-DNA-qPCR methods for assessment of chronic HBV infection in clinical samples

| **Sample No.** | **HBV-RNA-qPCR^a^ (copies/mL)** | **HBV-DNA-qPCR^b^ (copies/mL)** | **CRISPR-HBV-pgRNA assay** | |
| --- | --- | --- | --- | --- |
|  |  |  | **AuNP-LFB** | **RTF** |
| Test 1 | 2.65 × 10^2^ | 2.28 × 10^2^ | + | + |
| Test 2 | 4.62 × 10^3^ | 5.73 × 10^2^ | + | + |
| Test 3 | 3.27 × 10^5^ | 8.92 × 10^4^ | + | + |
| Test 4 | 1.57 × 10^5^ | 6.15 × 10^4^ | + | + |
| Test 5 | 6.27 × 10^3^ | 4.32 × 10^4^ | + | + |
| Test 6 | 1.37 × 10^4^ | 9.57 × 10^3^ | + | + |
| Test 7 | 3.54 × 10^4^ | 8.36 × 10^3^ | + | + |
| Test 8 | 7.69 × 10^5^ | 5.94 × 10^4^ | + | + |
| Test 9 | 1.32 × 10^5^ | 6.71 × 10^5^ | + | + |
| Test 10 | 6.82 × 10^2^ | 2.57 × 10^3^ | + | + |
| Test 11 | 3.27 × 10^5^ | 9.85 × 10^5^ | + | + |
| Test 12 | 5.72 × 10^3^ | 1.38 × 10^4^ | + | + |
| Test 13 | 3.67 × 10^4^ | 9.24 × 10^3^ | + | + |
| Test 14 | 5.67 × 10^2^ | 1.89 × 10^2^ | + | + |
| Test 15 | 3.57 × 10^2^ | 1.83×10^2^ | + | + |
| Test 16 | 9.82× 10^4^ | 3.54× 10^3^ | + | + |
| Test 17 | 1.37× 10^2^ | 1.13× 10^3^ | + | + |
| Test 18 | 3.27× 10^2^ | 5.76× 10^2^ | + | + |
| Test 19 | 2.78 × 10^3^ | 6.34 × 10^2^ | + | + |
| Test 20 | 9.67 × 10^4^ | 9.73 × 10^3^ | + | + |
| Test 21 | 9.74× 10^1^ | **—** | + | + |
| Test 22 | 3.58 × 10^4^ | 2.17 × 10^3^ | + | + |
| Test 23 | 5.67 × 10^4^ | 9.57 × 10^3^ | + | + |
| Test 24 | 3.81 × 10^4^ | 6.37 × 10^2^ | + | + |
| Test 25 | 8.19 × 10^5^ | 4.36 × 10^4^ | + | + |
| Test 26 | 3.95 × 10^3^ | 2.57 × 10^3^ | + | + |
| Test 27 | 7.34 × 10^4^ | 3.68 × 10^4^ | + | + |
| Test 28 | 8.26 × 10^2^ | 2.94 × 10^3^ | + | + |
| Test 29 | 3.24 × 10^2^ | 6.33 × 10^1^ | + | + |
| Test 30 | 2.97 × 10^4^ | 2.57 × 10^3^ | + | + |
| Test 31 | 9.57 × 10^4^ | 3.89 × 10^4^ | + | + |
| Test 32 | 7.34 × 10^3^ | 4.67 × 10^3^ | + | + |
| Test 33 | 2.68 × 10^4^ | 6.17 × 10^3^ | + | + |
| Test 34 | 8.65 × 10^4^ | 6.91 × 10^3^ | + | + |
| Test 35 | 2.89 × 10^3^ | 9.17 × 10^2^ | + | + |
| Test 36 | 5.43 × 10^3^ | 8.36 × 10^2^ | + | + |
| Test 37 | 9.26 × 10^3^ | 7.93 × 10^3^ | + | + |
| Test 38 | 5.67 × 10^4^ | 2.92 × 10^2^ | + | + |
| Test 39 | 2.91 × 10^2^ | 9.41 × 10^1^ | + | + |
| Test 40 | 1.84 × 10^4^ | 3.67 × 10^3^ | + | + |
| Test 41 | 1.16 × 10^2^ | 8.15× 10^1^ | + | + |
| Test 42 | 2.41 × 10^2^ | 9.34 × 10^1^ | + | + |
| Test 43 | 1.84 × 10^2^ | 8.56 × 10^1^ | + | + |
| Test 44 | 6.34 × 10^4^ | 2.85 × 10^3^ | + | + |
| Test 45 | 1.37 × 10^2^ | 5.69 × 10^1^ | + | + |
| Test 46 | 7.37 × 10^1^ | **—** | + | + |
| Test 47-70 | **—** | **—** | **—** | **—** |

**Notice:** ^a^HBV-RNA-qPCR, the HBV-RNA-qPCR diagnosis was performed using commercially available real-time TaqMan PCR Kit for HBV RNA (SanSure Biotech; Changsha, China). The concentrations of HBV-RNA >50 copies/mL were regarded as positive based on the manufacturer’s recommendations; ^b^HBV-DNA-qPCR, the HBV-DNA-qPCR diagnosis was performed using commercially available real-time TaqMan PCR Kit for HBV DNA (SanSure Biotech; Changsha, China). The concentrations of HBV-DNA >5 IU (~30 copies/mL) were regarded as positive based on the manufacturer’s recommendations.

+, Positive; —, Negative.


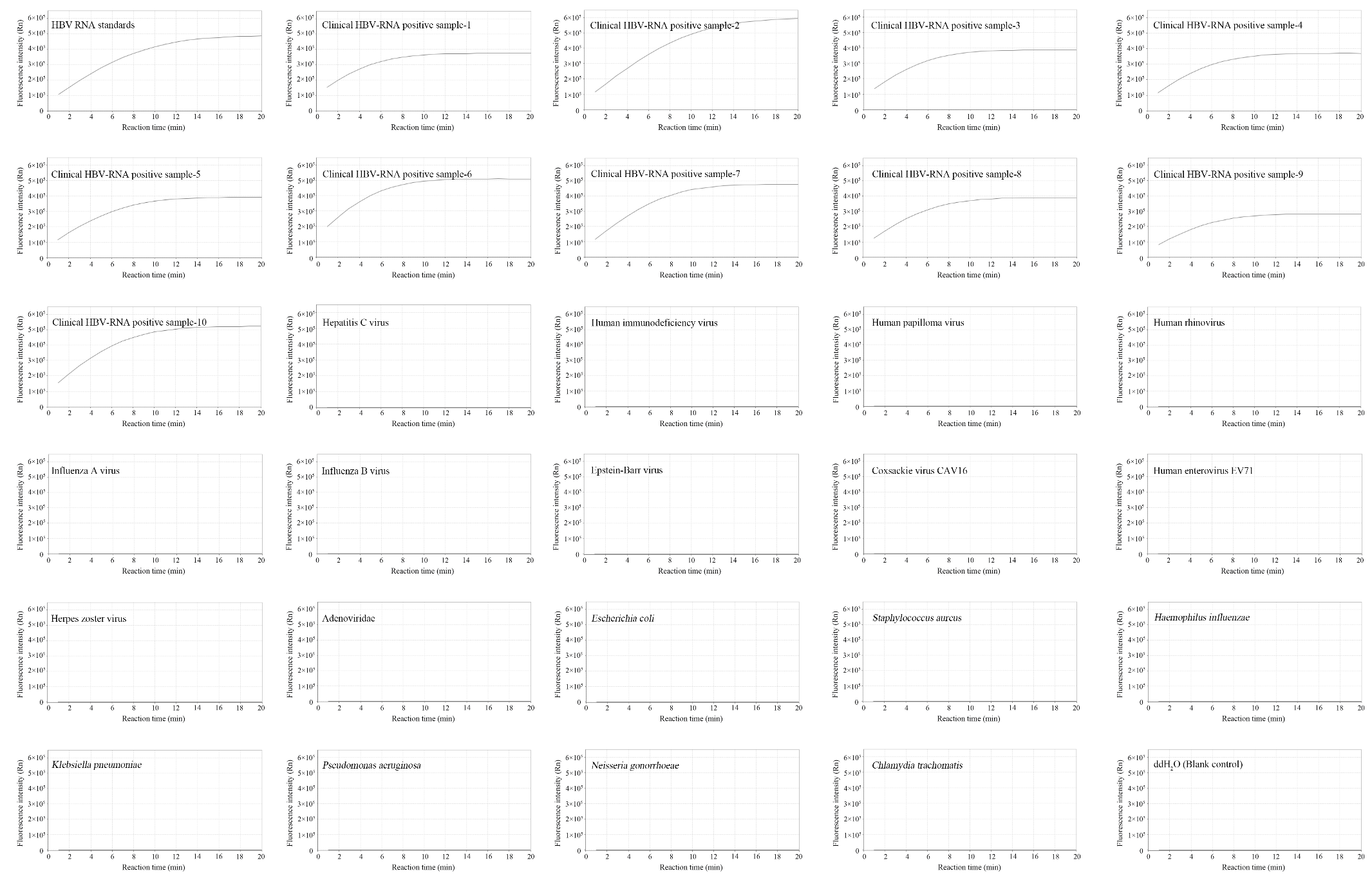


**Fig. S1 The specificity of CRISPR-HBV-pgRNA-RTF assay for HBV-pgRNA identification**

The LAMP amplification and CRISPR-Cas12b-based real-time fluorescence detection as described above. The HBV-RNA standard substance, HBV-RNA positive clinical serums (confirmed with HBV-RNA real-time qPCR) have been used as positive control. No signal was detected in other pathogens and blank control (ddH_2_O).

**
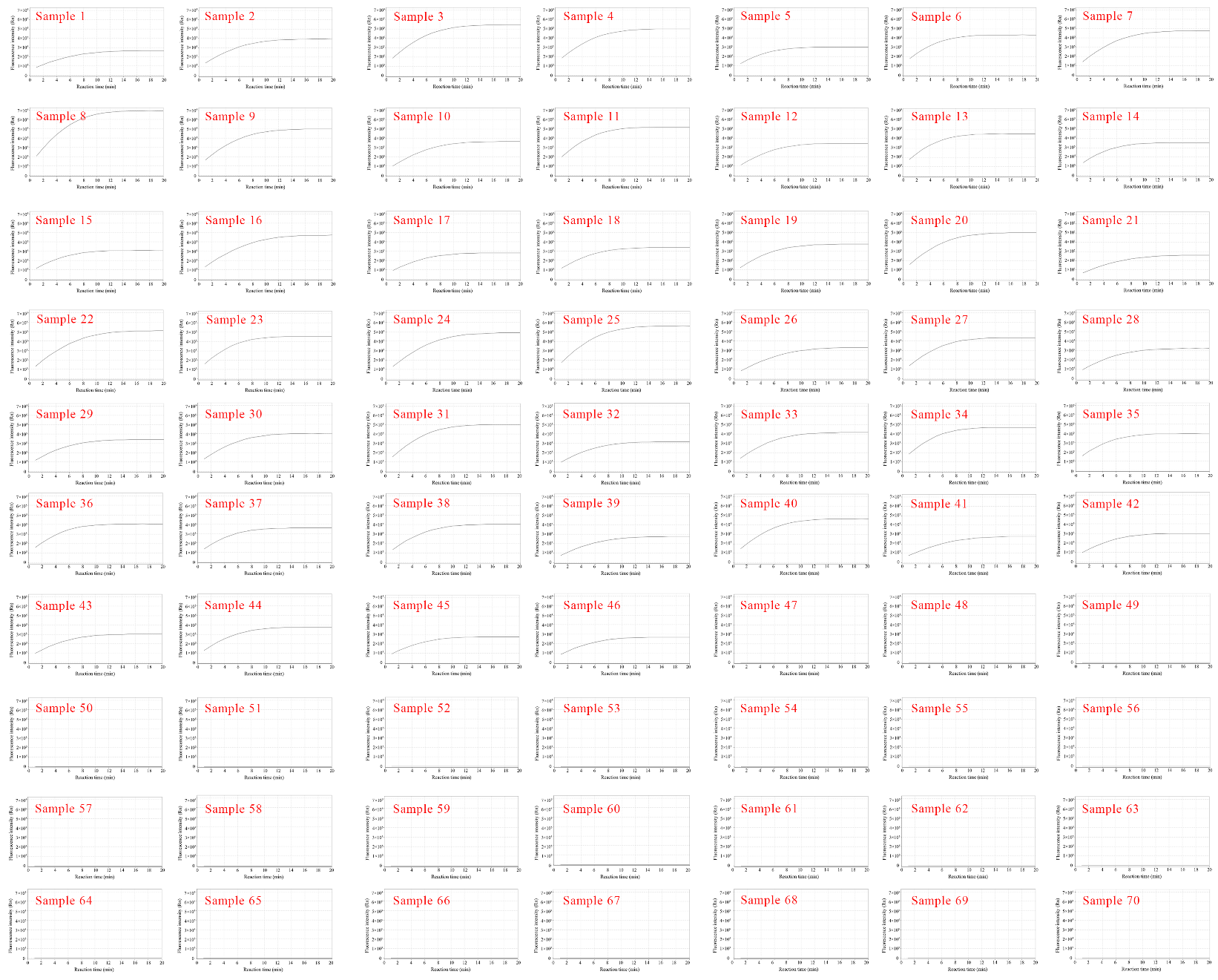
**

**Fig. S2 The results of CRISPR-HBV-pgRNA-RTF assay in clinical specimens**.

46 serum specimens from CHB patients (sample 1 to 46) and 24 serum specimens from healthy donors (sample 47 to 70) were tested through CRISPR-HBV-pgRNA-RTF assay. The results revealed that all 46 CHB samples (sample 1 to 46) were diagnosed as positive outcomes, and the other 24 serum specimens from healthy donors (sample 47 to 70) were tested as negative results.

**
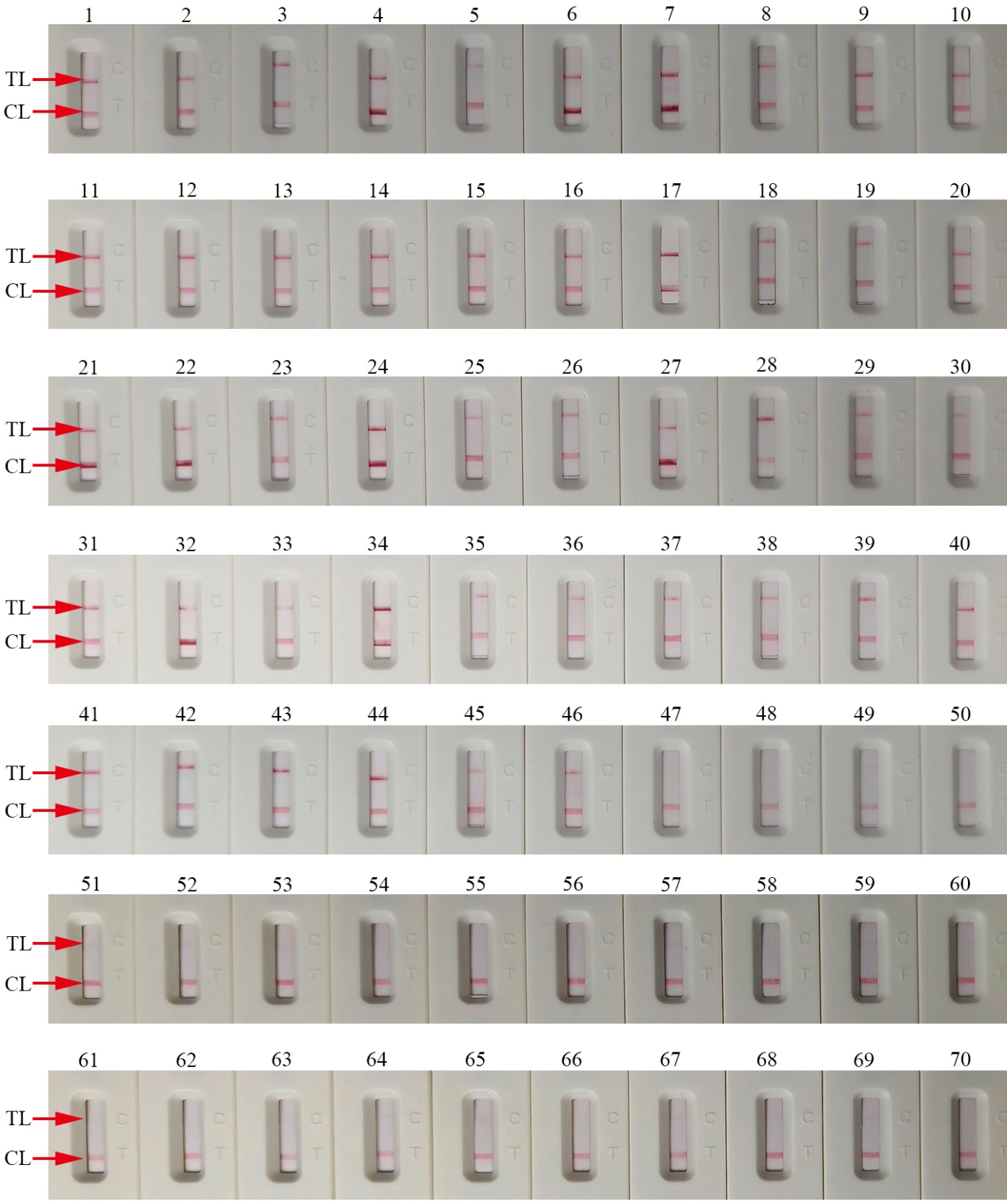
**

**Fig. S3 The results of CRISPR-HBV-pgRNA-AuNP-LFB assay in clinical specimens.**

46 serum specimens from CHB patients (sample 1 to 46) and 24 serum specimens from healthy donors (sample 47 to 70) were tested through CRISPR-HBV-pgRNA-AuNP-LFB assay. The results showed that all 46 CHB samples (sample 1 to 46) were diagnosed as positive outcomes, and the other 24 serum specimens from healthy donors (sample 47 to 70) were tested as negative results.
