# Supplementary figures and images for: Nanoparticle-based biosensor integrated with CRISPR/Cas12b platform for sensitive and visual identification of hepatitis B virus pregenomic RNA in chronic hepatitis B patients
Source: BMC Microbiol. 2026 Mar 24;26:485. doi: 10.1186/s12866-026-04900-4 (PMC13196004; doi:10.1186/s12866-026-04900-4)

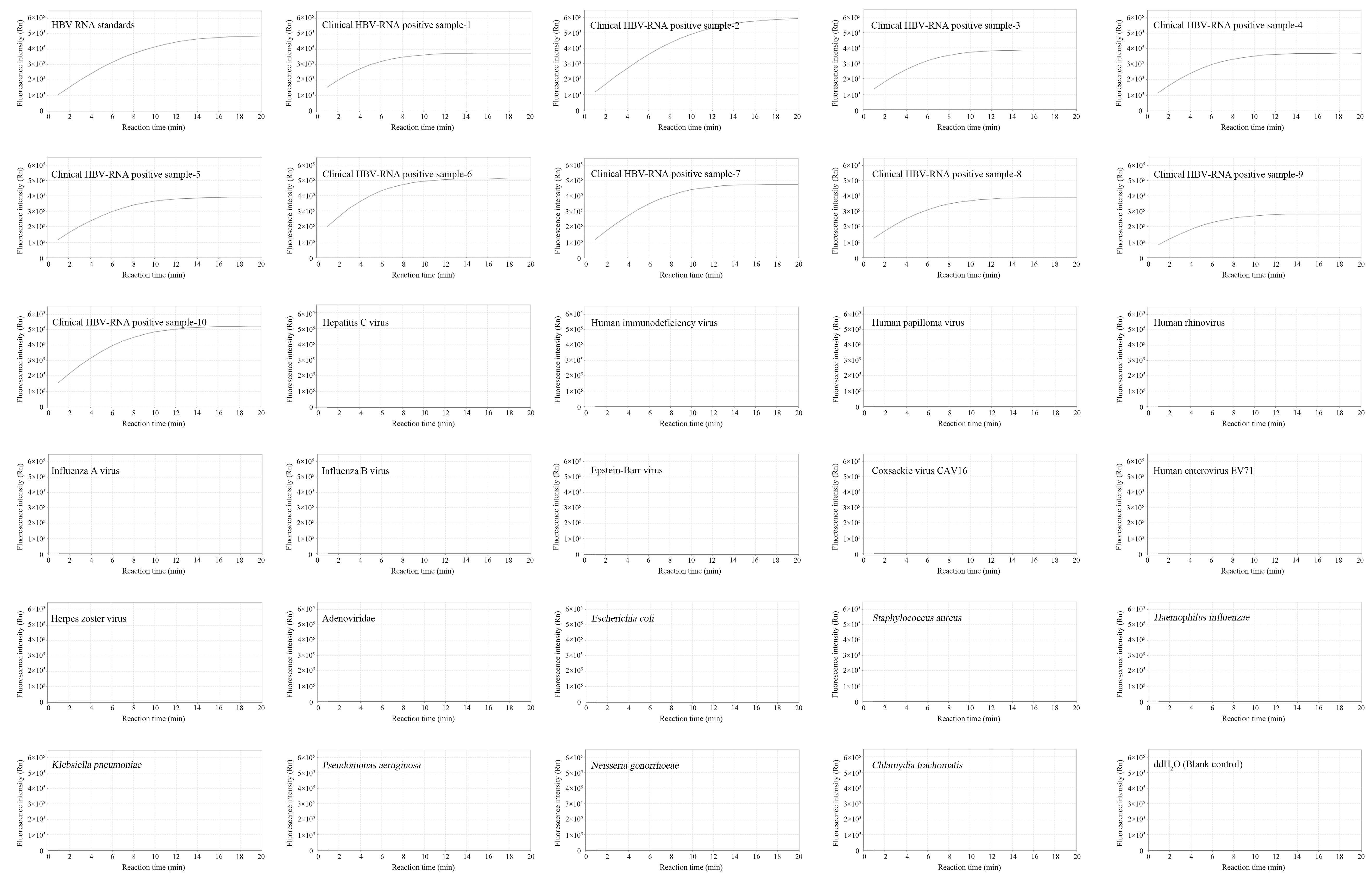

Supplement: Supplementary file 2 — Supplementary Material 2. [file 12866_2026_4900_MOESM2_ESM.tif]

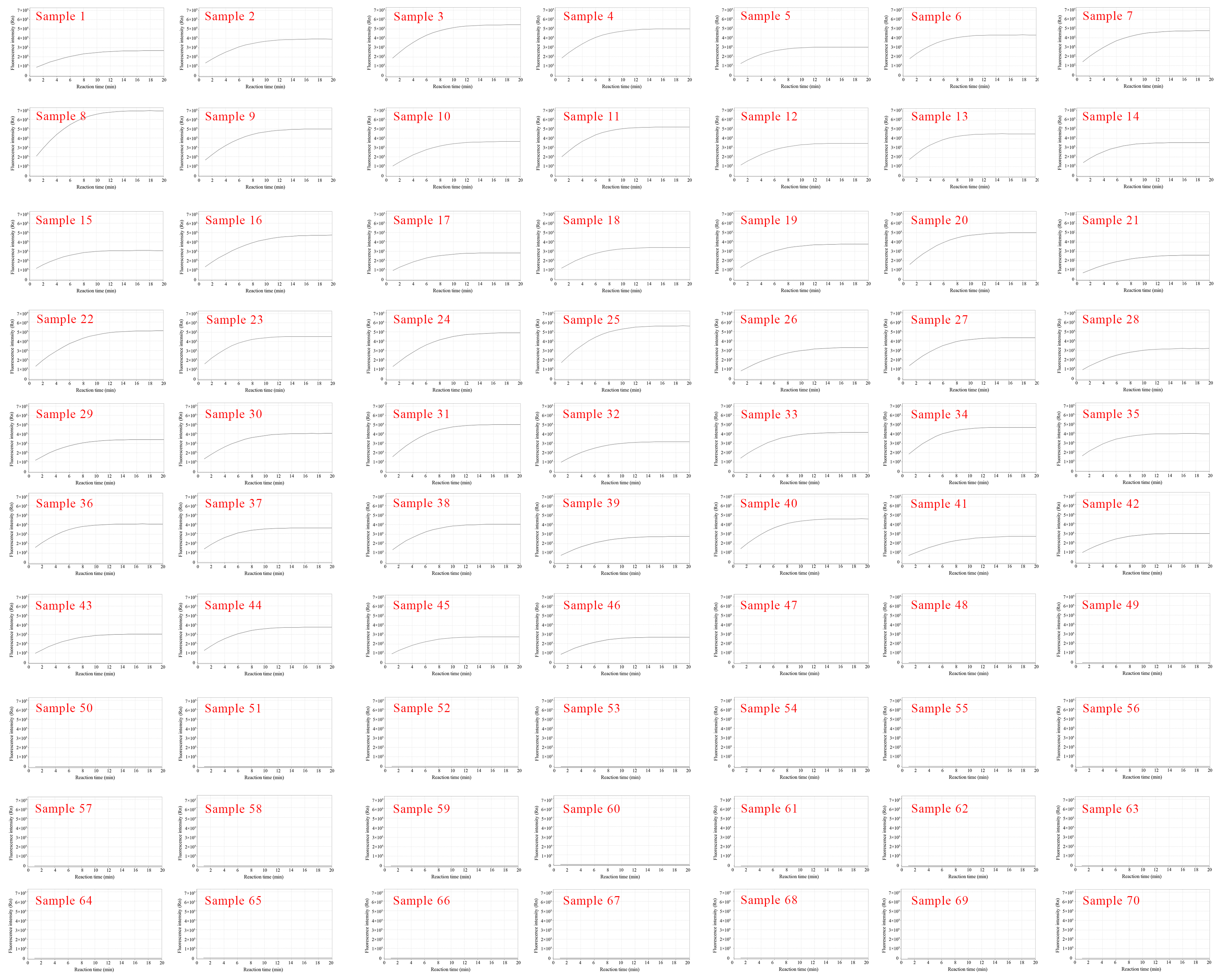

Supplement: Supplementary file 3 — Supplementary Material 3. [file 12866_2026_4900_MOESM3_ESM.tif]

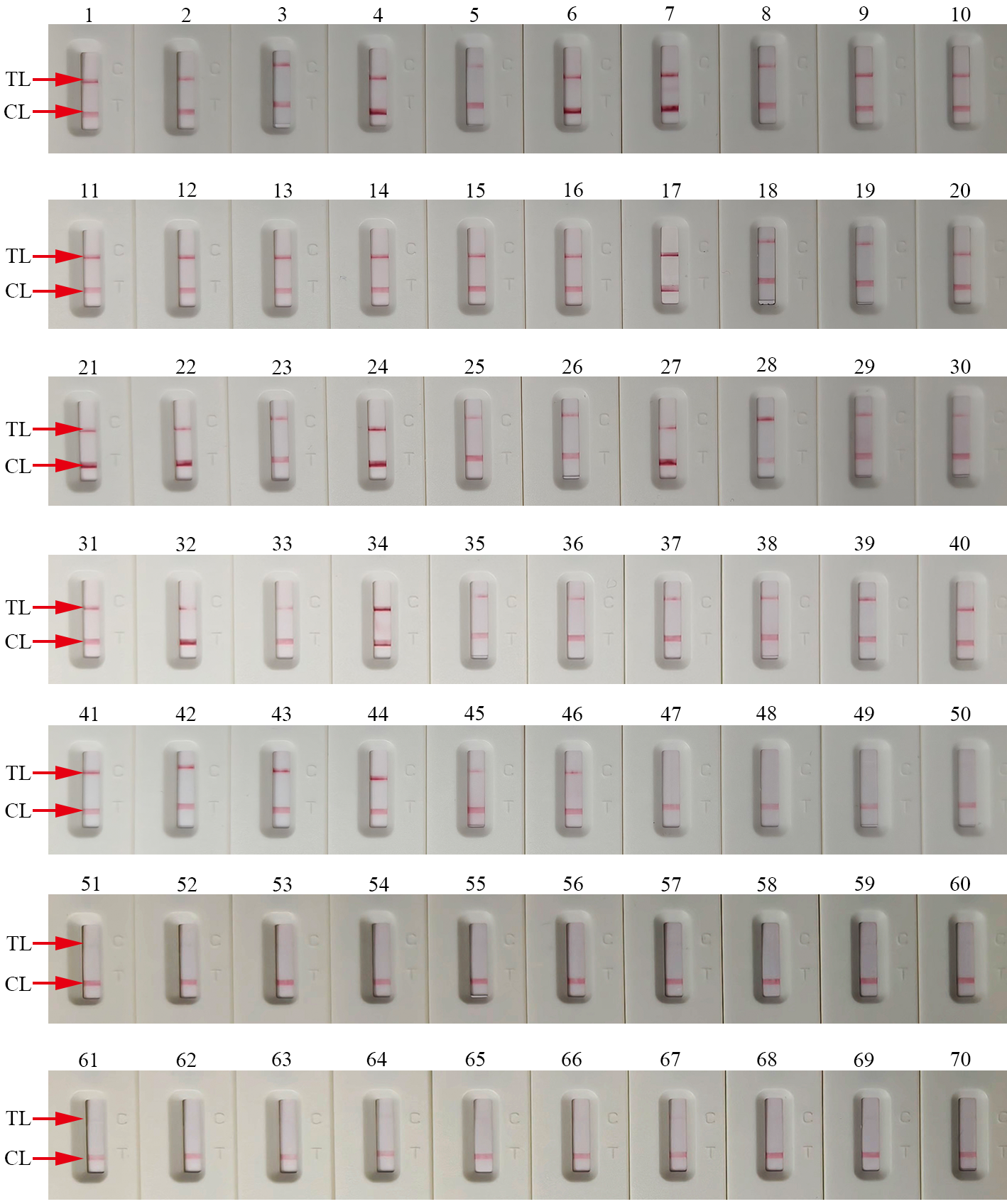

Supplement: Supplementary file 4 — Supplementary Material 4. [file 12866_2026_4900_MOESM4_ESM.tif]
