# Supplementary material for: Nanoparticle-based biosensor integrated with CRISPR/Cas12b platform for sensitive and visual identification of hepatitis B virus pregenomic RNA in chronic hepatitis B patients
Source: BMC Microbiol. 2026 Mar 24;26:485. doi: 10.1186/s12866-026-04900-4 (PMC13196004; doi:10.1186/s12866-026-04900-4)
Supplement: Supplementary file 5 — Supplementary Material 5. [file 12866_2026_4900_MOESM5_ESM.pdf]

贵州中医药大学第二附属医院伦理委员会伦理审查批件

Ethics Committee of The Second Affiliated Hospital of Guizhou University of Traditional Chinese Medicine Approval Notice

<table>
<tr><td>项目名称
Protocol name</td><td colspan="4">基于恒温扩增技术对 HBV 和 HCV 病毒检测方法的建立与应用研究
Identification of HBV and HCV using isothermal amplification platform</td></tr>
<tr><td>项目来源
Protocol source</td><td colspan="4">贵州省科技支撑计划项目（黔科合支撑[2023] 一般 242）
The Guizhou Provincial Key Technology R&D Program (Grant No. Qian Ke He Support [2023] General 242)</td></tr>
<tr><td>申请审查类别
Type of review</td><td colspan="4">☼初始 Initial review
☐修正后的重审 Review after revised</td></tr>
<tr><td>项目类别
Protocol type</td><td colspan="4">☐新药试验 New drug test：☐I 期 Phase-I ☐II期 Phase-II
☐III期 Phase-III ☐IV 期 Phase-IV
☐新器械或医用耗材试验 New instrument or medical supplies test
☼新技术试验 New technology test
☐临床研究 Clinical research
☐其他 Other（请注明 please indicate）：Case report</td></tr>
<tr><td>伦理批件号
Ethical Apprpval NO.</td><td colspan="4">KYW 2022/034</td></tr>
<tr><td rowspan="4">项目主要研究者
基本信息
Basic information on Principal investigaor of The project</td><td>姓名
Name</td><td>陈旭
Xu Chen</td><td>学历
Degree</td><td>博士研究生
Doctor</td></tr>
<tr><td>科室
Department</td><td>临床医学实验室
Clinical Medical Laboratory</td><td>职务
Title</td><td>副主任
Vice Director</td></tr>
<tr><td>职称
Technical Post</td><td>副教授
Associate Professor</td><td>电话
Telephone</td><td>0851-85514432</td></tr>
<tr><td>邮箱
E-mail</td><td>xuchen1220@126.com</td><td>手机
Mobile phone</td><td>18275185692</td></tr>
<tr><td rowspan="3">科研处/医务处
审查意见
Review opinion of the Scientific Research Division/Department of Medical Administration</td><td colspan="2">项目相关资料是否齐全
Project related data
Are complete</td><td colspan="2">☼是 Yes
☐否 No</td></tr>
<tr><td colspan="2">审查专家
Review expert</td><td colspan="2">[illegible] [illegible]</td></tr>
<tr><td colspan="4">审批意见（Approval comments）：
[illegible]
办公室主任签字（Signed）：
[illegible]
日期 Date：2022.3.8
贵州中医药大学第二附属医院 医学伦理委员会</td></tr>
</table>
